# New General Bacterial Stain

**Published:** 1917-04

**Authors:** 


**New General Bacterial Stain.** Munoz Uvra, Rev. Vallisoletana de Especialides, No. 11, 1916, describes the method as follows: 1. Dry and fix in flame; 2. Stain with 1% aqueous solution of methylene blue and pass through the flame; 3. Wash; 4. Wash for ½ minute in a cold solution containing to each 100 c.c., 6 drops of sulphuric acid and potassium bicarbonate to saturation; 5. Wash in water for 1 minute; 6. Apply to the mount a few drops of 1:500 safranine in water; 7. After 1 minute wash and dry. Mount in colophane dissolved in gasoline. Bacteria are stained deep blue or violet, the nuclei show all details, the cellular substance is rose-violet and intercellular connections are dark gold color.

